# The Effect of Boron Nitride on the Thermal and Mechanical Properties of Poly(3-hydroxybutyrate-co-3-hydroxyvalerate)

**DOI:** 10.3390/nano8110940

**Published:** 2018-11-15

**Authors:** Mualla Öner, Gülnur Kızıl, Gülşah Keskin, Celine Pochat-Bohatier, Mikhael Bechelany

**Affiliations:** 1 Chemical Engineering Department, Chemical-Metallurgical Faculty, Yildiz Technical University, Istanbul 34210, Turkey; kizilgulnur@gmail.com (G.K.); gulsahkeskin9@gmail.com (G.K.); 2 Institut Européen des Membranes, IEM UMR-5635, ENCSM, CNRS, Université de Montpellier, ENSCM, CNRS, Place Eugéne Bataillon, 34000 Montpellier, France; celine.pochat@umontpellier.fr

**Keywords:** biopolymer, bionanocomposite, poly(3-hydroxybutyrate-co-3-hydroxyvalerate), boron nitride, mechanical properties, thermal properties

## Abstract

The thermal and mechanical properties of poly(3-hydroxybutyrate-co-3-hydroxyvalerate, PHBV) composites filled with boron nitride (BN) particles with two different sizes and shapes were studied by scanning electron microscopy (SEM), differential scanning calorimetry (DSC), X-ray diffraction (XRD), Fourier Transform Infrared Spectroscopy (FTIR), thermal gravimetric analysis (TGA) and mechanical testing. The biocomposites were produced by melt extrusion of PHBV with untreated BN and surface-treated BN particles. Thermogravimetric analysis (TGA) showed that the thermal stability of the composites was higher than that of neat PHBV while the effect of the different shapes and sizes of the particles on the thermal stability was insignificant. DSC analysis showed that the crystallinity of the PHBV was not affected significantly by the change in filler concentration and the type of the BN nanoparticle but decreasing of the crystallinity of PHBV/BN composites was observed at higher loadings. BN particles treated with silane coupling agent yielded nanocomposites characterized by good mechanical performance. The results demonstrate that mechanical properties of the composites were found to increase more for the silanized flake type BN (OSFBN) compared to silanized hexagonal disk type BN (OSBN). The highest Young’s modulus was obtained for the nanocomposite sample containing 1 wt.% OSFBN, for which increase of Young’s modulus up to 19% was observed in comparison to the neat PHBV. The Halpin–Tsai and Hui–Shia models were used to evaluate the effect of reinforcement by BN particles on the elastic modulus of the composites. Micromechanical models for initial composite stiffness showed good correlation with experimental values.

## 1. Introduction

Plastics are the preferred materials in many areas of daily life since they are easily processed, low-cost, light, and durable. Approximately 7% of the world’s oil and natural gas is used for the production of plastics. Beside limited fossil resources, widespread usage of these nondegradable materials leads to very serious environmental problems [1,2]. The development of commercially viable bioplastics is an attractive alternative to nondegradable polymers with their renewable resources and biodegradability [3]. Polyhydroxyalkanoates (PHAs) came into prominence among biodegradable polymers synthesized by many bacteria as intracellular carbon and energy storage granules. They have very good properties such as versatility, high biodegradability in proper environmental conditions, and similar mechanical performance with petroleum-based polymers such as polypropylene (PP) [4].

Poly(3-hydroxybutyrate) (PHB) is a linear bacterial polyester. It is among the most known and the best characterized member of PHA family [5]. It is a highly crystalline polyester, is very brittle and has a very low biodegradation rate because of its high crystallinity (>90%). One solution to this problem is to copolymerize PHB with 3-hydroxyvalerate (HV) monomers in the bacterial fermentation process to form poly(3-hydroxybutyrate-co-3-hydroxyvalerate) (PHBV). The addition of HV units improves the mechanical properties, increases the thermal stability and prevents the degradation during processing. PHBVs can be obtained with different properties depending on the percentage of HB and HV units. The excess of HV units leads to the polymer being in a softer and ductile form and having a lower crystallinity value [6,7]. PHBV has a high potential application in many areas such as medical and agriculture fields and as packaging material. However, widespread applications of PHBV are still hindered by several material drawbacks such as high material costs, slow crystallization rate, poor thermal stability, brittleness and relative difficulty in processing. Recently, the addition of nanofillers and fibers as reinforcing agents into polymer matrixes to form nanocomposites has provided a promising method because they can act as nucleating agents, not only improving the polymer crystallization rates but also increasing the mechanical, thermal and/or barrier performances of the composites. Nanofillers are found to be preferable in many applications due to their high surface area-to-volume ratios and low concentrations needed to achieve reinforcing effects [8,9,10].

PHBV is a highly crystalline polyester with low degree of heterogeneous nucleation density. It is not thermally stable around the melting point. Molecular weight reduction can be observed during the melting process. In order to enhance the performance of PHBV the effect of various nanofillers on crystallization and mechanical behaviors has been investigated [11]. Chen et al. worked on structural and mechanical properties of PHBV/OMMT (organo-montmorillonite) nanocomposites [12]. The tensile stress of nanocomposites improved up to 32% for 3% (wt) OMMT content compared with the neat polymer. Optimum mechanical properties were obtained for 3% (wt) OMMT content. Further increasing of filler addition leads to agglomeration of OMMT causing the decrease of mechanical properties of composites [12]. Ten et al. studied the thermal and mechanical properties of PHBV/cellulose nanowhiskers biocomposites [13]. The tensile strengths of the composites have increased due to the strong interface linkages between the nanoparticles and the polymer. The tensile strength of 5 wt.% cellulose nanocomposite improved by 35.5% compared to pure PHBV, while the toughness value increased by 41% [13]. Choi et al*.* produced cloisite 30B organic clay-filled PHBV nanocomposites by the solution intercalation method [14]. Compared to neat PHBV, improvements in mechanical properties were observed in nanocomposites with 1, 2% and 3 wt.% cloisite 30B content [14]. Öner et al. worked on PHBV/hydroxyapatite (HAP) composites [15]. The mechanical properties of PHBV were improved using HAP particles. Xiang et al. studied the mechanical properties of PHBV/green tea polyphenol (TP) composites [16]. The results showed that the elongation at break, toughness, strain and tensile stress of composite increased with TP addition when compared to pure PHBV [16]. Luo and Netravali obtained green composites by using PHBV and pineapple fibers [17]. The flexural strength and modulus of the obtained composites, in the longitudinal direction, increased with fiber loading [17]. Nanocellulose-reinforced PHBV was prepared by Jun et al. [18]. They investigated the effect of nanocellulose types such as cellulose nanocrystals (CNC) and cellulose nanofibrils (CNFs) on mechanical properties of PHBV. The maximum tensile modulus values were obtained for 7 wt.% CNC and CNF composites but the tensile stresses of composites are lower than the tensile stress of neat PHBV [18]. The ternary cellulose/PHBV/polylactic acid (PLA) composite was developed to compromise the 100% degradability of materials [19]. Filling PHBV/PLA blends with the ball-milled celluloses increased the stiffness when using different particle sizes and filling contents [19]. The tensile strength, flexural strength and compressive strength of the composite were improved by mixing PLA fiber with PHBV [19]. Ternary nanocomposites including cellulose nanocrystals/silver nanohybrids (CNC-Ag) and biodegradable poly(3-hydroxybutyrate-co-3-hydroxyvalerate) (PHBV) were prepared by using solution casting [20]. Compared to binary PHBV/CNC nanocomposite, the ternary nanocomposites with the highest AgNPs content, showed the largest improvement in the thermal stability, mechanical, barrier, overall migration and antibacterial properties [20].

2D nanomaterials have recently been a very active research area due to their small thicknesses, wide lateral surfaces, and weak Van der Waals interactions between layers. Wang et al. produced (PHBV)/graphene nanosheet (GNS) composites via a solution-casting method and investigated their mechanical properties. The results showed that the storage modulus of PHBV/GNS composites highly improved with GNS addition [21]. Recently, research activity has increased in the area of boron nitride (h-BN) nanomaterials. Boron nitride has a layered structure, where Van der Waal’s forces hold sheets of covalently bonded boron and nitrogen atoms together. The hexagonal form of BN (h-BN) is similar to graphene which accounts for their high thermal conductivity. The hexagonal boron nitride nanosheets are of great interest due to their potential use in various real-life applications. They are popular fillers for polymers due to unique material properties [22,23,24]. Zhi et al. studied the fabrication of boron nitride nanosheets and production of polymeric composites with improved thermal and mechanical properties [25]. BN was exfoliated via ultrasonication and used as filler for PMMA/BN nanocomposites. The thermal expansion coefficient and glass transition temperature were reduced in composites compared with the neat polymer. These results indicated that polymer chain mobility reduced due to BN nanosheet-matrix interactions. Young’s modulus of PMMA was improved by 22% and strength was increased by 11% with the addition of 0.3% (wt) BN [25]. Wattanakul et al. investigated the effect of sonication and dissipation of BN on the mechanical properties of epoxy/BN nanocomposites. Impact strength of composites improved up to 33% (v) BN content and started to decrease at 37% (v) BN content. Filler particles tend to stay as agglomerates in high filler contents. Thus, the decrease of mechanical properties could be observed in high filler contents [26]. Pradhan et al. studied the effect of BN particles on mechanical properties of starch. The tensile stress of nanocomposites improved up to 3 times for 10% BN content compared to neat starch [27]. Cheewawuttipong et al. studied on the polypropylene (PP)/BN composites. The mechanical analysis results showed that the storage modulus and loss modulus increased with BN content [28]. Sun et al. combined fused silica (FS) with BN to improve mechanical properties. The flexural strength and toughness values increased significantly with 0.5 wt.% BN addition [29]. Chitosan/Boron nitride (BN) composites were prepared by solution method with variable percentage of boron nitride loading. It was found that, the thermal stability of the chitosan/BN composites was increased in comparison to virgin chitosan [30]. Zhou et al. investigated the effect of BN nanoparticles on mechanical properties of epoxy matrices. Tensile modulus increased from 2.68 ± 0.21 GPa to 3.14 ± 0.31 GPa for 50 wt.% BN loading. However, the mechanical strength, toughness, and elongation at break (%) values decreased with increasing BN content [31]. Polyamide 6 (PA6) and BN and exfoliated BN(BNNS) composites were produced by Li et al. [32]. The tensile stress of PA6/BN composites was higher than neat PA6 but for BNNS composites, the greater enhancement was obtained due to the higher aspect ratio BNNS and interaction between the polymer and BNNS [32]. The reinforcement effect of graphene-like BN on the gelatin was investigated by Biscarat et al. [33]. The barrier properties of gelatin/BN nanocomposites have been enhanced by a factor of 500 compared to a neat gelatin.

In this study, we investigated the effect of BN on mechanical and thermal properties of PHBV. This research is in the continuity of our study on the improvement of the properties of PHBV by incorporating boron nitride particles with a twin-screw extruder so that it could be transferred to industry for large-scale production. In our previous work, h-BN were studied as the potentially interesting material for the enhancement of barrier properties of PHBV [34,35]. Based on our preliminary results, the aim of this work was to investigate the mechanical and thermal properties of PHBV composites by taking into consideration the effect of different BN nanoparticles. In order to develop this understanding, polymer nanocomposites containing boron nitride nanoparticles of two different shapes, hexagonal disk (OSBN) and nanoflakes (OSFBN) were prepared through melt processing route with different concentrations. Various techniques analyzing mechanical and thermal properties were employed to characterize the PHBV/BN nanocomposites. The best mechanical properties are obtained for the nanocomposite sample containing 1 wt.% of the silanized flake type BN (OSFBN) for which an increase in Young’s modulus up to 19% was observed in comparison to the neat PHBV. The resulting biobased and biodegradable PHBV/BN nanocomposites may find potential applications in the fields of packaging and biomedical devices.

## 2. Materials and Methods

### 2.1. Materials

PHBV, the biopolymer with 8 mol% hydroxyvalerate (HV) content was purchased from ADmajoris Company, Cublize, France under the trade name MAJ’ECO FN000HA. Two different types of hexagonal nano-sized BN were used. One of the BN’s was purchased from Bortek, Eskisehir, Turkey (2.27 g/cm^3^, surface area 20 m^2^/g); the other BN (FBN), commercial grade (PHPP325B) (2.2 g/cm^3^, surface area 60 m^2^/g), from Saint-Gobain Ceramics, France. Octyltriethoxysilane (OTES) was purchased from Sigma-Aldrich, Steinheim, Germany.

### 2.2. Surface Modification of Boron Nitride

The surface modification of boron nitride was performed by using the silanizing agent to produce an appropriate interface between matrix and filler. BN (1.5% w/v) particles were added to the 90:10 (v/v) ethanol-water mixture and treated with an ultrasonic probe system (Sonic vibra cell VCX 750, Newtown, CT, USA) for 30 min with an amplitude of 40%. The mixture was then centrifuged (Sigma 3-16P) at 4000 rpm for 35 min. Octyltriethoxysilane (OTES) with a concentration of 2.5% (w/v) was dissolved in a 90:10 (v/v) ethanol-water mixture and the pH of the solution was adjusted to 5.0 using dilute HCl solution. The solution was stirred by an ultrasonic mixer for 2 h at room temperature for silane hydrolysis. BN particles were added to the solution and sonicated with an ultrasonic probe for 30 min with an amplitude of 40%. The solution was centrifuged at 4000 rpm for 10 min. Finally, the obtained silanized BN was dried at 110 °C in an oven for 2 h and then at 65 °C in a vacuum drier.

### 2.3. Preparation of Nanobiocomposites

Nanobiocomposites were prepared by melt-mixing method. Double screw extruder (D: 10 mm, L/D: 20, Rondol, UK) was used to obtain PHBV/BN nanocomposites. Before extrusion, both of the polymer and boron nitride were dried at 50 °C for an hour in a vacuum dryer to remove the moisture. Extruder temperatures from the feed zone to the endpoint have been applied 90-135-160-160-150 °C, respectively. The rotation speed of the screws is 80 rpm. Polymer nanocomposite films prepared using a hot-cold press machine (Gülnar Makine, İstanbul, Turkey). The mechanical analysis specimens were cut to 2 mm thickness and dumbbell shape in accordance with ISO 527-1BA standard. The prepared nanocomposites were given in Table 1. OS code shows the silanized samples.

### 2.4. Characterization of BN Nanoparticles and Composites

X-Ray diffraction analyses were collected on a PHILIPS X’pert Pro Panalytical diffractometer, Egham, Surrey, UK (2θ = 2–80°, 40 kV, 20 mA, λ = 1.54 Å) in order to investigate crystalline structure of nanocomposites. The analysis was performed at room temperature. FTIR analysis was performed by using BRUKER Alpha-P, (Coventry, UK) in the 400–4000 cm^−1^ region. Scanning Electron Microscopy (SEM) was carried out by using the instrument FEI-Philips XL 30 ESEM-FEG (Amsterdam, The Netherlands) in order to investigate the morphologies of samples and the dispersion of BN particles in composites. Particle sizes were found using ImageJ software.

### 2.5. Thermal Properties of PHBV/BN Nanobiocomposites

In order to investigate the thermal properties of PHBV and PHBV/BN composites, DSC measurements were performed on TA Instruments (DSC Q20 V24.11 Build 124, New Castle, DE, USA). The analysis was carried out in three steps at a heating and cooling rate of 10 °C/min in an aluminum crucible under 50 mL/min nitrogen atmosphere. In the first heating step, samples of 10 mg mass were heated from 0 °C to 200 °C at a rate of 10 °C/min and kept at this temperature for 2 min to erase thermal history of the material. Then the samples were cooled from 200 °C to 0 °C at a cooling rate of 10 °C/min (cooling run) and kept at this temperature for 2 min. Then, the samples were re-heated to 200 °C at a rate of 10 °C/min. The melting and the crystallization temperatures (T_m_ and T_c_) as well as the melting and the crystallization enthalpies (ΔH_m_ and ΔH_c_) were determined. The crystallinity was calculated from the formula below [34]:

(1) $$\;\chi_{C}\left(\%\right)=\left[\frac{\Delta H_{m}}{\left(W_{PHBV}\times \Delta H_{m}^{ref}\right)}\right]\times 100\;$$

where $\Delta H_{m}$ is the melting of sample, $W_{PHBV}$ is the weight fraction of PHBV in the composite and $\Delta H_{m}^{ref}$ is the theoretical melting enthalpy for 100% crystallized PHBV, 146 J/g [34].

TGA analysis was performed using a thermogravimetric analyzer (TA Instruments, Q500 V 20.13 Build 39, New Castle, DE, USA). About 10 mg sample was weighed and analyzed in a platinum crucible by heating at a heating rate of 10 °C/min up to 800 °C under a 40 mL/min nitrogen-60 mL/min air environment.

### 2.6. Mechanical Properties of PHBV Nanobiocomposites

Uniaxial tensile testing was performed according to ASTM D882-12 standard, by using 2 kN capacity Devotrans (161070 CKS GP, Istanbul, Turkey) mechanical testing machine. Specimens were kept at 50 °C in a ventilated oven for 48 h for conditioning before the test. Mechanical analyses of nanocomposites were performed using 5 mm/min tensile rate and 1 N preload at room temperature. Tensile strength at break, Young’s modulus, and elongation at break values were determined from the stress-strain curves. Five specimens of each sample group were tested, and the average results were reported.

## 3. Results and Discussion

### 3.1. Morphological Characterization of BN Particles by SEM

**Figure 1:**
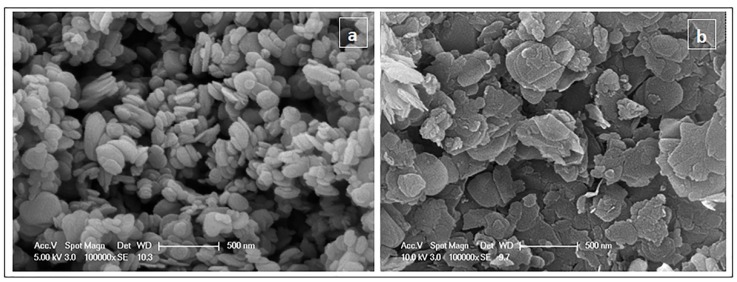
Scanning Electron Microscopy (SEM) micrograph of particles after 60 min ultrasonication (**a**) silanized BN (**b**) silanized FBN.

The morphology and the size of the BN particles were examined by scanning electron microscopy (SEM). Figure 1 compares the SEM images of silanized OSFBN and OSBN particles after 60 min ultrasonic treatment. The OSFBN particles showed predominantly irregular flake-type shaped morphology whereas, in case of the OSBN particles, the predominant shape was nearly hexagonal disk particles as shown in Figure 1a,b. We examined SEM micrographs of several samples by using Image J software and performed statistical analysis on the particle sizes. The results show that the size of the particles was reduced after ultrasonic treatment. The mean length (L), the mean width (w) and thickness (t) of the OSFBN particles were reduced from 2445.9 ± 1507.6 nm to 765.4 ± 376.8 nm; from 1483.7 ± 853.8 nm to 360.49 ± 177.8 nm and from 249.7 ± 137.4 nm to 19.0 ± 5.1 nm, respectively. The mean diameter (d) and thickness (t) of OSBN particles, reduced from 225.0 ± 108.0 nm to 163.3 ± 72.9 nm and from 61.6 ± 25.8 nm to 39.7 ± 10.7 nm, respectively after ultrasonication. The standard deviations given here describe the variation in the mean value calculated from separate SEM images. The range of values was on the order of hundreds of nanometers, indicating the polydispersity of each particle system.

### 3.2. SEM of Nanocomposites

**Figure 2:**
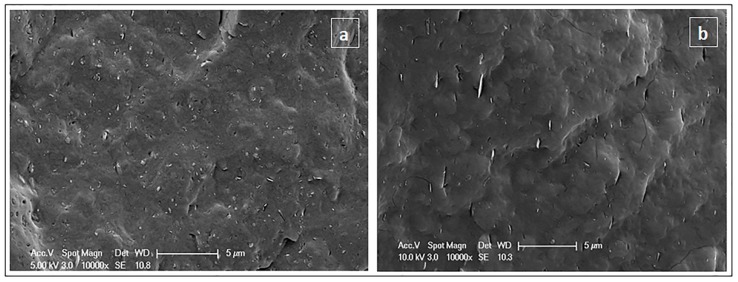
SEM images of composites (**a**) PHBV/1OSBN (**b**) PHBV/1OSFBN.

The structure of the PHBV/BN nanocomposites was investigated using SEM to get a qualitative understanding of the dispersion of BNs through direct visualization. Figure 2 shows the cross-section SEM images of the cryo-fractured surfaces of 1 wt.% PHBV/OSBN and PHBV/OSFBN composite samples. The dispersion of boron nitride in composites can be seen in the SEM figures. A good dispersion of the BN particles in the matrix was observed at 1 wt% loading.

### 3.3. Characterization by XRD and FTIR

**Figure 3:**
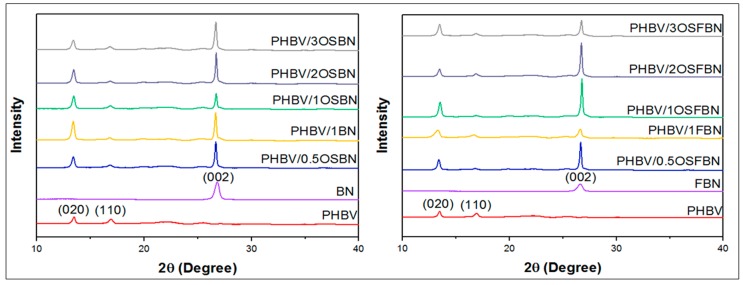
XRD patterns of neat PHBV, BN, FBN and nanocomposites.

**Figure 4:**
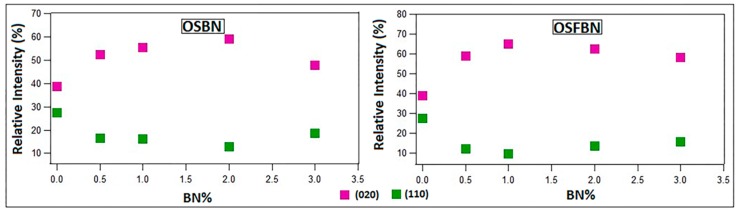
(020) and (110) relative intensity variation with BN content.

XRD analyses were carried out to investigate the crystalline structure of prepared nanocomposites (Figure 3). The XRD patterns of PHBV exhibited characteristic 2θ peaks at 13.6° (020), 17.1° (110), 19.9° (021), 21.7° (101), 22.3° (111), 25.5° (121), 27.1° (040) and 30.3° (002) [36,37]. Boron nitride exhibited characteristic 2θ peaks at 26.80° (002). Nanocomposites show same reflections as neat PHBV indicating that boron nitride incorporation did not change the unit cell and crystalline structure of PHBV. However, the intensity of (020) and (110) peaks of PHBV changes with BN content as shown in Figure 4. While the intensity of (020) peak of PHBV increases, the intensity of (110) peak of PHBV becomes lower with boron nitride addition. The increase of (020) peak of PHBV could be related to the crystallite lamella size of polymer and indicated that crystallization is promoted by boron nitride addition [38]. The decrease of (110) peak of PHBV indicates the restricted crystal growth in (110) plane. (020)/(110) relative intensity ratios for PHBV and nanocomposites were given in Table 2. While the relative intensity ratio of the neat PHBV matrix was 1.42, this value increased up to 4.58 for the OSBN nanocomposites and 6.72 for OSFBN nanocomposites. The increase in the relative ratio implies that crystal growing was promoted in (020) crystal plane as reported [38,39].

**Figure 5:**
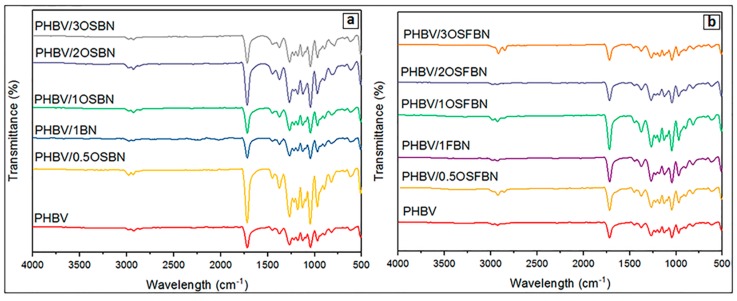
FTIR spectrum of nanocomposites (**a**) PHBV/BN (**b**) PHBV/FBN.

**Figure 6:**
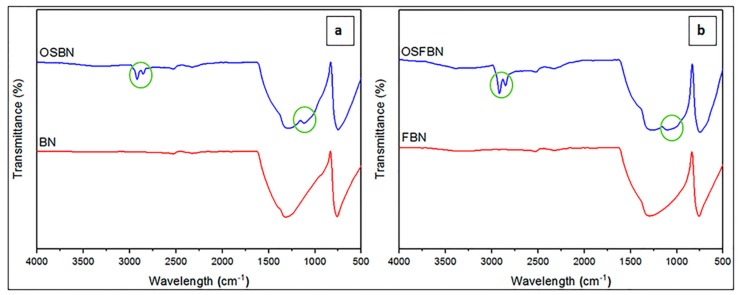
FTIR peaks of silanized (**a**) BN and (**b**) FBN.

Figure 5 shows the FTIR spectra of neat PHBV and the prepared nanocomposites. PHBV exhibited some characteristic peaks as CH_3_ asymmetrical stretching at 3015–2960 cm^−1^, CH_2_ asymmetrical stretching at 2945–2925 cm^−1^, CH_3_ symmetrical stretching at 2885–2865 cm^−1^, C=O stretching at 1723–1740 cm^−1^, CH_2_ wagging at 1320–1159 cm^−1^ [40], asymmetrical –C–O–C– stretching, symmetrical –C–O–C– stretching at 800–975 cm^−1^ [41], CH_2_ scissoring at 1453–1459 cm^−1^, C–O stretching at 1065–1030 cm^−1^ and C–C stretching at 979–980 cm^−1^ [42]. BN exhibited characteristic peaks as B–N at 1300–1400 cm^−1^ and B–N–B at 775–820 cm^−1^ [43,44]. OTES exhibited Si–O stretching at 1053–1114 cm^−1^ and CH_n_ (C–H) stretching at 2850–3000 cm^−1^ [45,46]. The silane peaks were not observed in FTIR spectrum of composites because of overlapping of silane peaks with PHBV peaks in the same region. Figure 6 shows a comparison between the infrared spectrum of silanized and nonsilanized BN particles. After both BN particles were treated with silane, the spectrum showed new bands in addition to the characteristic peaks of BN. In the spectrum of OSBN and OSFBN, the bands at 2850–3000 cm^−1^ regions were attributed to the CH_2_ asymmetric and symmetric stretching vibration, respectively, which originated from the silane-containing molecule. The band at 1053 cm^−1^ is assigned to the in-plane Si–O stretching and that at 1114 cm^−1^ is assigned to the perpendicular Si–O stretching.

### 3.4. Thermal Stability of Nanocomposites

**Figure 7:**
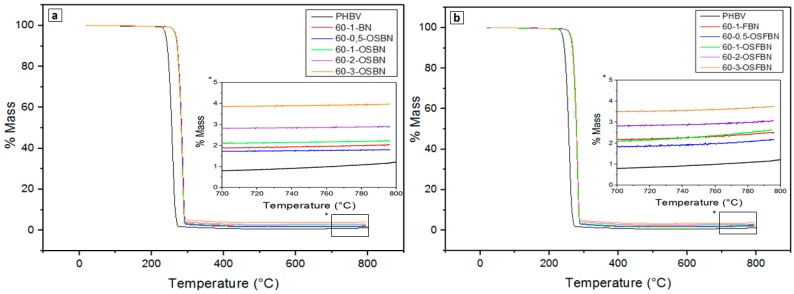
TG thermograms of (**a**) PHBV/OSBN and (**b**) PHBV/OSFBN nanocomposites.

The thermal stability of the PHBV and PHBV/BN composites were studied using Thermo Gravimetric Analysis (TGA) to measure the degradation temperature of different samples. The TGA thermograms for neat PHBV was compared with that of the PHBV/BN nanocomposites. TGA curves of PHBV and composite samples are represented in Figure 7. It is noted that weight loss of PHBV and its composites occurs in a one-step process between 230 °C and 300 °C. No significant weight loss was recorded before 200 °C for all samples. After 200 °C, weight loss proceeds very rapidly and the polymer completely degrades by 300 °C. It has been established that the thermal degradation of PHBV was due to the rupture of ester bonds during chain scission [47]. This could be attributed to PHBV copolymer separating out into individual PHV and PHB units. The temperatures of at 10% weight loss (T_10_), at 50% weight loss (T_50_), the initial decomposition temperature (T_i_), and the maximum rate of degradation temperature (T_max_) are presented in Table 3. As shown in Table 3, T_i_, T_10_, T_50_ and T_max_ values increased in the composites. When the initial weight loss is taken as a point of comparison, the onset degradation temperature (T_i_) for neat PHBV is 234.45 °C and increases to 252.70 °C and 251.10 °C for PHBV/1OSBN and PHBV/1OSFBN composites. The surface treatment by silane results in improved initial thermal stability in comparison to untreated BN. Another important thermal property is the temperature corresponding to the maximum rate of weight loss (T_max_). T_max_ shifted to higher temperatures as the BN content increased, from 275.08 °C to 295.50 °C. This result showed that the thermal stability of the composites improved with the addition of the BN to the polymer matrix. One of the most important property of boron nitride is its high-temperature resistance. As a result of this property, inclusion of the BN within a polymeric matrix results in increasing of the thermal stability of the composite. The fabrication of gelatin-BN nanocomposites was investigated by Biscarat et al. [33]. An increase of gelatin degradation temperature was observed by using DSC. It was concluded that gelatin chains that intercalate into BN are restricted by the nanosheets, and the movement of segments is restrained. An electrostatic interaction or a hydrogen bond between the charged groups of gelatin chains and BN acts as physical crosslinking and reduces the activity of the gelatin [33].

Camargo et al. investigated thermal behavior of PHBV/Lignin composites by thermogravimetric analysis and found that the thermal decomposition of pure PHBV and composites took place in a single degradation step [48]. In another study, Bhardwaj et al. worked on the thermogravimetric analysis of PHBV/cellulose fibers [49]. Cellulose fibers did not affect the existing degradation step of PHBV. Lai et al. studied the thermal properties of multilayered carbon nanotube/PHBV composites [50]. It was observed that the degradation temperature rise was up to 16 °C for the PHBV nanocomposite. It was concluded that nanodispersion of carbon nanotubes increased thermal stability of composites [50].

### 3.5. Thermal Properties of Composites

**Figure 8:**
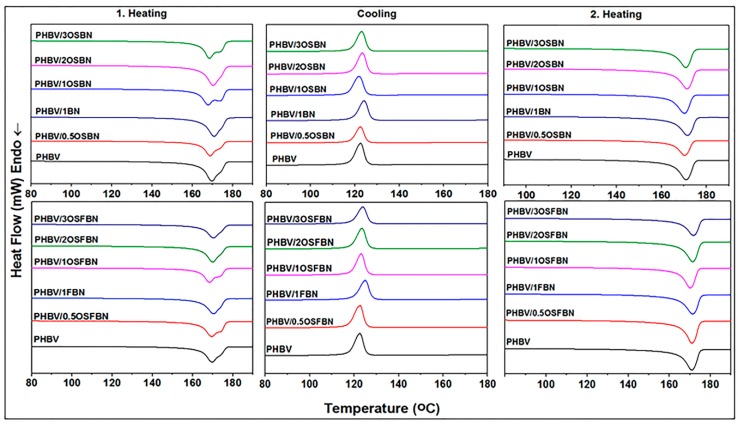
DSC thermograms of PHBV/BN and PHBV/FBN nanocomposites.

Differential scanning calorimetry (DSC) was used to study the change in enthalpy values associated with chemical phase transitions in composite samples, as a function of temperature. Two heating and one cooling cycles were performed in order to make useful comparisons for PHBV/OSFBN and PHBV/OSBN composites. Figure 8 and Table 4 present the thermograms obtained from the cooling, first and second heating cycle at 10 °C/min for PHBV and PHBV/BN composites. The various thermal property results calculated from the DSC heating and cooling curves are summarized in Table 4. As can be observed from Figure 8, neat PHBV and PHBV/BN composites showed two melting peaks during the first heating. The peak maximum temperatures of first and the second melting peaks are given in Table 4 as T_m1_ and T_m2_. The double melting endotherms have been reported by several groups [51,52,53]. The double melting peak in polymers may be due to several reasons. The origin for the double melting behavior of PHBV is still being researched. It was generally accepted that the double melting peaks were caused by melting–recrystallization–melting behavior during heating scans [52]. The first melting peak values are in the range of 166–171 °C for composites as opposite to 170 °C for neat PHBV. The degree of crystallinity (Xc) from the first heating scan was computed and presented in Table 4. The BN addition does not influence crystallinity of the matrix for silanized samples up to 3 wt% loadings. The addition of the surface treated BN to the polymer matrix (PHBV/1OSBN) slightly increased the crystallinity of PHBV from 57% to 60%.

The thermograms and data obtained from the second heating cycle provide information on the crystallization and melting behavior of the samples without the influence of different thermal histories. The broad or double melting peaks observed during the first heating cycle were not present in the second heating cycle. Table 4 shows the degree of crystallinity (%) of PHBV and PHBV/BN composites measured during the second heating cycle. In general, it was observed that the degree of crystallinity was higher when measured during the second heating cycle compared with that measured during the first heating cycle. The addition of BN increased the degree of PHBV crystallinity from 68% to 70% and 71% for PHBV/2OSBN and PHBV/1OSFBN composites respectively. Further increasing of the BN concentration to 3% decreased the crystallinity values of the composites. This behavior can be explained by the particle dispersion and distribution in the PHBV matrix. When the particles are dispersed well, the higher surface area helps the particles to act as nucleating agent. The change in particle size due to aggregation and the surface characteristics of the particle leads to the decrease in crystallinity. Similar to the first heating cycle, the addition of treated BN increased the crystallinity of PHBV. The crystallinity of PHBV/BN composites treated with silane was slightly higher than that displayed by composites without any silane treatment except for sample PHBV/3OSBN.

The crystallization temperature (T_c1_) and the heat of crystallization (ΔH_c_) were determined from the DSC cooling runs of samples. These are shown in Table 4 for composites with different BN contents. From Table 4, cold crystallization temperature nanocomposites did not much change with the change in nanoparticle concentration. The heat of crystallization of 1 wt.% PHBV/1OSBN and PHBV/1OSFBN composites was higher than the neat PHBV, while it decreased with the increase in nanoparticle loading. This behavior can be explained by agglomeration of BN particles at higher loadings.

In a study by Sanchez Garcia et al., Tm values of PHBV nanocomposites did not change or reduced slightly [54]. There are many factors that affect the melting temperature, such as molecular order, crystal thickness, and crystal perfection. Polymer degradation due to melt processes and chain separation can cause this small decrease, but other factors are also influential [54]. Hassaini et al. investigated thermal properties of PHBV/olive husk flour (OHF) composites by DSC [55]. They found that the melting temperatures of PHBV did not change with OHF addition, however, the melting enthalpies increased. It shows that OHF filler contributes to the crystallization of PHBV as a nucleating agent. The effect of graphene nanoplatelets (GNPs) on the mechanical properties of high-density polyethylene (HDPE) nanocomposites was investigated [56]. The crystallinity of the HDPE composite was decreased by increasing the concentration of GNPs as a result of the formation of the smaller crystalline domains in HDPE in the presence of nanomaterials. Crystal defects in the presence of inhomogeneities were believed to decrease the matrix crystallinity [56]. Covalent bonds across the interface have been shown to increase crystallinity whereas attractive noncovalent interactions have shown decreasing or unchanging in crystallinity in comparison to the neat polymer [57,58]. Yu et al. reported that the crystallinity of the electrospun fibers of PHBV decreases when adding ZnO [59]. This result was explained by the decrease of the PHBV crystallinity decreased because of the formation of hydrogen bonds between PHBV and ZnO.

### 3.6. Mechanical Analysis Results

**Figure 9:**
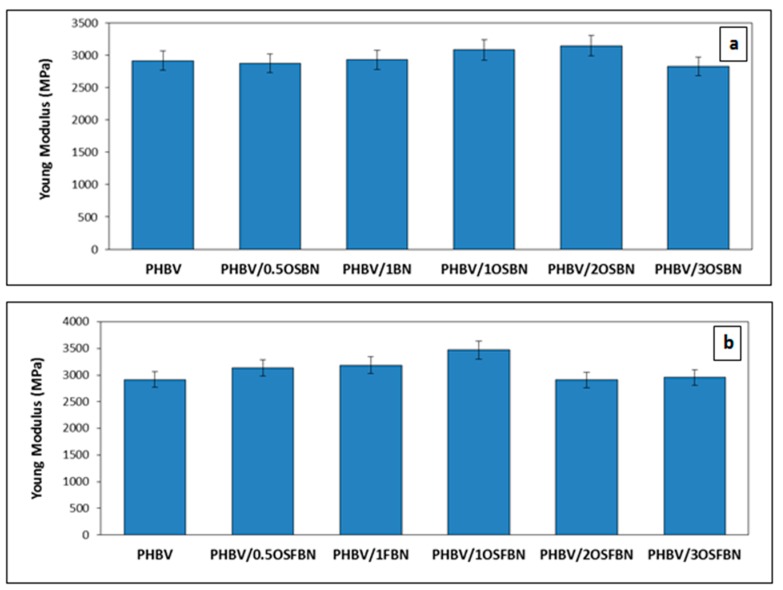
Young’s modulus values of (**a**) PHBV/BN and (**b**) PHBV/FBN nanocomposites.

**Figure 10:**
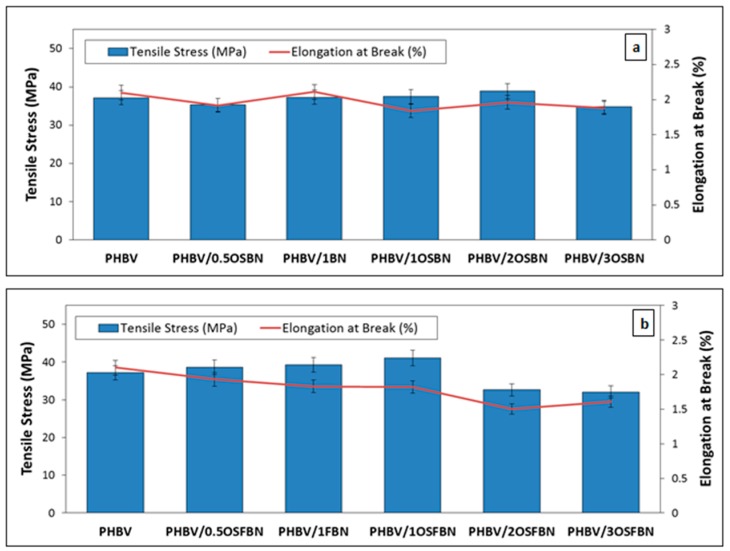
Tensile stress and elongation at break values of (**a**) PHBV/BN and (**b**) PHBV/FBN nanocomposites.

There are many factors that influence the mechanical properties of nanocomposites. The vast majority of these factors depend on the properties of the fillers such as size and aspect ratio, distribution and orientation of the particles. The main difference between traditional fillers and nanofillers is that nanosize materials have a much wider surface (interface) per unit volume [60]. Tensile tests are applied in order to investigate the effect of BN addition and silanization on mechanical properties. Properties such as tensile modulus, tensile strength, and strain-at-break were measured as shown in Figure 9 and Figure 10. As shown in Figure 9, adding 0.5 wt% OSBN essentially had no effect on the modulus. PHBV with 2 wt% OSBN had the highest value of modulus. It increased as well the neat PHBV stiffness by 8%. The addition of 3 wt% OSBN decreased the composite Young’s modulus and the tensile strength. The addition of OSBN did not have a significant impact on the tensile strength of the composites. Furthermore, the elongation at break of neat PHBV decreased with increased OSBN loading. Maximum elongation decreased, from 2.1% for neat PHBV to 1.9% with the addition of 3 wt% OSBN. The slight increase in strength and the decrease in elongation in the composites might be attributed to an embrittlement caused by BN agglomeration.

Both the Young’s modulus and tensile strength of PHBV/OSFBN composites were increased for films containing 1 wt% OSFBN content. The addition of nanocrystals caused enhancement of the Youngs modulus and the tensile strength up to 1 wt% but led to a decrease at higher loadings. Young’s modulus for the PHBV/1OSFBN composite was found to be around 3469.7 MPa which accounts for a maximum 19% increase. The maximum increase of the tensile strength of PHBV/1OSFBN composite is 10.6%. The elongation at break of PHBV decreased with the addition of OSFBN. This result shows that BN filler particles were well dispersed at low filler loadings but nonhomogenously distributed at higher concentrations.

**Figure 11:**
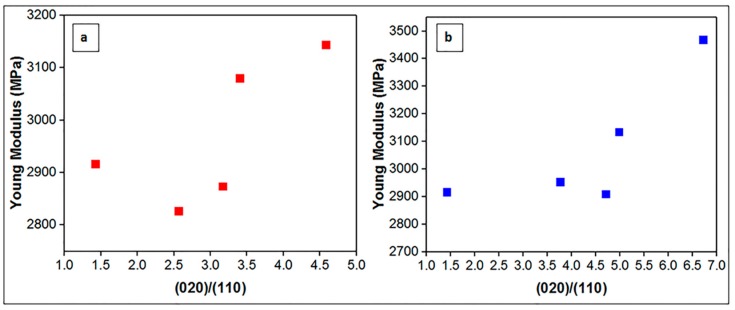
Young’s modulus variation with (020)/(110) relative intensity ratio of (**a**) PHBV/OSBN and (**b**) PHBV/OSFBN composites

These findings are in agreement with the XRD results. Figure 11 shows the Young’s modulus of the composites as a function of (020)/(110) relative intensity ratio for neat PHBV and PHBV/OSBN and PHBV/OSFBN nanocomposites. As seen from the Figure 11, all composites had higher (020)/(110) ratio than the neat PHBV. 1 wt.% PHBV/OSFBN composite samples showed the highest (020)/(110) ratio and the highest Young’s modulus compared to other composites as well as neat PHBV. This was consistent with the decrease in the (020)/(110) ratio of 3 wt.% PHBV/BN samples. Young’s modulus of 3 wt.% PHBV/BN composite samples showed the lowest (020)/(110) ratio. This result associated to XRD analyses shows that BN filler particles at low filler loadings directly influence the morphological organization of PHBV polymer matrix and increase the stiffness of the polymer. However, the effect of nanoparticles addition is not linear and above a certain limit there is a reduction of stiffness enhancement. Maximizing the properties of the polymer at low nanoscale in loadings of a nanoscale filler requires that it is thoroughly distributed throughout the polymer matrix and that complete exfoliation of the filler’s layers has occurred.

For the same BN content, the OSFBN composites had higher tensile strength and modulus than OSBN composites. These results showed that the mechanical properties of the composites were found to increase more for the PHBV/OSFBN compared with PHBV/OSBN samples. The higher values in strength and modulus observed in the PHBV/OSFBN specimen compared with the PHBV/OSBN can be attributed to different factors: (1) OSFBN was dispersed more uniformly in the composite specimens than the OSBN; (2) this may be related to the higher surface area displayed by OSFBN (71.90 m^2^/g), which can promote a better intercalation of BN nanosheets between PHBV chains in comparison to OSBN (26.89 m^2^/g) and can influence the polymer chain organization. Studies concerning surface area have shown that reinforcement is related to nanoparticle surface area [61]. The larger surface area of OSFBN leading to the stronger interactions between the BN and PHBV. In this work, the partial exfoliation of the OSFBN allows for maximum surface area exposure between the filler and PHBV; (3) the flake-like nanoparticles showed higher tensile strength than disk type particles.

It is well known that the homogeneous dispersion of nanoparticles in the polymer matrix is necessary to improve properties of composite. The good dispersion of the both BN particles in the matrix was observed in SEM figures (Figure 1a,b). Apart from the distribution of the filler, understanding the effect of the shape and aspect ratio of the filler on the composite’s properties impacts its design. As we have observed in SEM images, the aspect ratio for OSFBN flakes are around 19 and OSBN disks be around 4. The property improvement of the OSFBN may be attributed the higher surface area and aspect ratio of the platelets.

To obtain a strong interface, the filler should have an attractive interaction with the matrix. The interaction may be achieved through surface chemistry in the form of functionalization with chemical moieties. In order to see surface functionalization on mechanical properties, the composite samples were prepared without using coupling agent. Young’ modulus values of nonsilanized nanocomposites with 1 wt.% BN and 1 wt.% FBN content are 2929 MPa and 3187 MPa respectively. After silanization, the modulus values increased to 3080 MPa and 3469 MPa, for OSBN and OSFBN nanocomposites respectively. This shows that good interfacial adhesion was achieved between PHBV and BN, which might be due to the silane coupling agent used in this study.

Carotenuto et al. prepared LDPE/GNP (low-density polyethylene/graphite nanoplatelets) nanocomposites and tested the mechanical performance. It was found that both tensile elongation at break and compressive extension were reduced in films containing GNP [62]. It was claimed that this is due to the obstruction in polymer chain mobility, where the polymer chains are not allowed to unfold and rotate when stress is applied due to the uniform presence of GNP throughout the LDPE matrix. Yu et al. prepared PHBV/cellulose nanocrystal-silver (CNC-Ag) nanocomposites and tested the mechanical performance of nanocomposites [36]. They obtained the highest Young’s modulus and the lowest elongation at break for 10% CNC-Ag content. These improvements have been shown to be a consequence of the homogeneous distribution of the CNC-Ag additive, the increased interfacial adhesion between two phases by means of interaction of hydrogen bonds, and the increased crystallization of PHBV. However, with further increase of CNC-Ag filler content to 15 wt.% or more let to the decrease in mechanical properties was observed. This result was explained by the agglomeration of the filler. Xiang et al. prepared PHBV/tannic acid nanocomposites and examined the mechanical performance. Mechanical properties such as tensile stress and fracture toughness were improved comparing to neat PHBV matrix. However, no improvement was observed above a certain level of filler content [63].

### 3.7. Mechanical Modelling

Multiple analytical, mechanics-based theories were developed to model particles-filled composite structures. These theories are reliant on filler volume fraction and elastic properties of each constituent. As a result, these models offer a good indication of the resultant properties but are unable to account for the effect of factors, such as particles interaction and distribution, on the properties of the composites. All models used for the calculation of relative modulus assumed perfect interfacial adhesion between particles and matrix. In this work, the experimental data obtained were compared with two of the simplest and most common theoretical models to predict Young’s modulus of the composite materials; Halpin–Tsai and Hui–Shia models. The Halpin–Tsai model accounts the modulus of reinforcement and matrix materials as well as shape and volume fraction of filler. The mechanical modeling of a variety of reinforcement of fillers such as platelet-like or flake-like fillers can be done by using this model [64]. The Halpin–Tsai equation for randomly oriented discontinuous fillers is given in Table 5 [65]. The Hui–Shia model is employed to the mechanical modeling of composites with the assumption of perfect interfacial bonding between the polymer matrix and fillers. The Hui–Shia equation for platelet fillers is given in Table 5. In the Hui–Shia model, α represents the aspect ratio of the filler, which is the width of the platelet divided by the thickness of particles. In this work, SEM images were used for calculation of aspect ratio. As observed by SEM, the shape of the OSBN nanoparticles was a nearly hexagonal disk with an aspect ratio of approximately 4.1. The OSFBN nanoparticles showed irregular flake-like shapes with the mean aspect ratio of 18.9. E_c_, E_m_ and E_f_ symbolize the Young’s modulus of nanocomposite, matrix and filler respectively. E_m_ value was taken from directly Admajoris as 2.95 GPa. E_f_, the Young’s modulus of hexagonal boron nitride was taken from literature as 40 GPa [24]. The factors $\eta_{L}$ and $\eta_{T}$ are given by equations in Table 5 as a function of E_f_ (the modulus of the filler) and E_m_ (modulus of the matrix). Φ_f_ symbolizes the volume fraction of nanoparticle in composite, it is defined as:

(2) $$\;\phi_{f}=\frac{V_{BN}}{V_{BN}+V_{PHBV}}\;$$

The volume fraction for composites with 0.5 wt.%, 1 wt.%, 2 wt.% and 3 wt.% BN content was calculated using this formula. In the Halpin–Tsai model, *ξ* which depends on the width (*w*) and thickness (*t*) of nanoparticles and symbolizes the shape factor is defined as:

(3) $$\;\xi =2\left(\frac{w}{t}\right)\;$$

In the Hui–Shia model, g is the geometrical parameter which depends on the aspect ratio of the filler. The parameters ξ and Λ are defined in equations given in Table 5.

**Figure 12:**
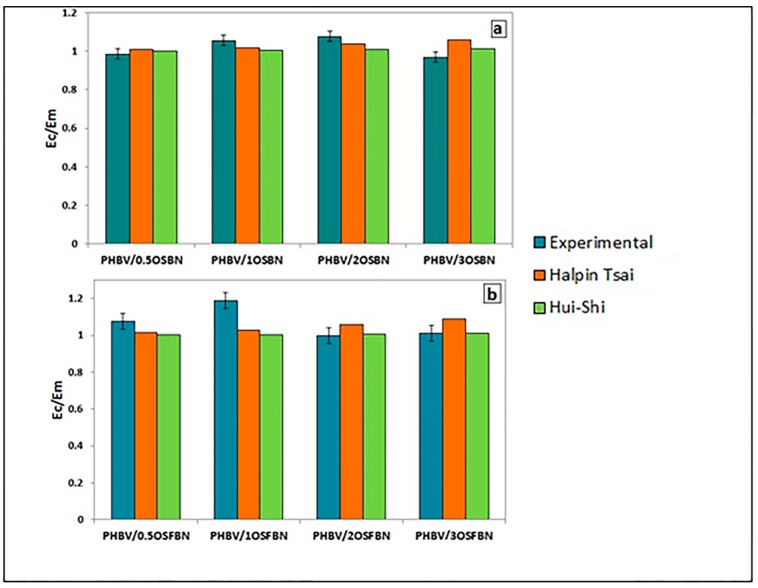
Comparison of mechanical models with experimental data for (**a**) PHBV/OSBN and (**b**) PHBV/OSFBN nanocomposites.

Figure 12 compares the model results predicted by the Halpin–Tsai and Hui–Shia empirical relations for the modulus of the PHBV/BN composites with the experimental results obtained from the tensile testing. Generally, theoretical modeling was in good agreement with experimental data. From the figure, the measured values of PHBV/OSFBN samples at low loadings are higher than predicted value of the models, but model equations displayed higher modulus values than the obtained experimental values at higher loadings. The models overestimate reinforcement phenomena at higher loadings. This may be due to several causes; the presence of agglomerations in the matrix weakening the structure, the inhomogeneous distribution of the BN reinforcement by extrusion in higher loadings, and the weak interaction between the nonpolar filler and the nonpolar matrix. Improvements in any of these three facets should result in moduli that are more accurately predicted by the models. On the other hand, both models are heavily reliant on filler volume fraction of the reinforcing phase. It should be noted that for rigid reinforcing phase, an increase in particle volume fraction often correlates to an increase in theoretical Young’s modulus. Both models have been shown to underestimate the potential of particles to reinforce polymers at lower loadings. This discrepancy may be attributed to the lack of model’s ability to predict reinforcement effect of particles at low loadings. The deviation (%) in E_c_/E_m_ of nanocomposites for different mechanical models is given in Table 6. Model predictions were in good agreement with experimental results.

## 4. Conclusions

PHBV nanocomposites containing BN nanoparticles with different sizes, shapes, and specific surface areas were processed and characterized to elucidate the effects of nanoparticles on nanocomposite properties. PHBV/BN nanocomposite processing which is scalable from the laboratory to an industrial setting was prepared via a masterbatch by using the twin-screw extruder. SEM morphology showed the good dispersion of BN in the PHBV matrix with using coupling agent at low loadings. The interaction between PHBV and boron nitride was evidenced by FTIR and XRD. TGA analysis showed that the thermal decomposition of PHBV was retarded by the interaction between BN and PHBV. DSC measurements did not show significant alterations to the crystalline content of PHBV. The matrix crystallinity was not significantly affected by the change in filler concentration but decreasing of the crystallinity of PHBV/BN composites was observed in higher loadings. The crystallinity of the PHBV was not affected by the differences in the nanoparticle shapes which indicated that the interfacial interactions between polymer and both particle systems were weakly attractive. The nanoparticles were likely to be located in the amorphous regions of the PHBV matrix as the crystalline content of polymer matrices did not change significantly with the increase in particle concentration.

BN increased the modulus of PHBV, as observed via tensile tests. However, elongation at break decreased as the amount of BN increased. The Young’s modulus of the composite was increased by 19% in PHBV/1OSFBN composite. At higher loading, BN agglomeration takes place within the PHBV matrix and the lack of proper adhesion between the matrix and the BN results in insufficient stress transfer. Moreover, modified BN has been shown to contribute to an enhancement of Young’s modulus than unmodified BN. The differences in the mechanical behavior of the two BN particles were attributed to the different surface area and aspect ratio characteristics. The higher surface area (71.9 m^2^/g) and aspect ratio (18.9) displayed by OSFBN can promote a better intercalation of BN nanosheets between PHBV chains and influence the mechanical properties. Theoretical modeling can be used to predict the potential modulus improvements of adding a filler to a matrix. In our research, The Halpin–Tsai and Hui–Shia models were used to evaluate the effect of reinforcement by BN particles on the elastic modulus of the resulting composites. Both model equations predict values close to the experimental results.

## Figures and Tables

**Table 1:** The prepared nanobiocomposites for mechanical analysis.

Sample	Boron Nitride Content (wt.%)
PHBV	-
BORTEK	
PHBV/0.5OSBN	0.5
PHBV/1BN	1
PHBV/1OSBN	1
PHBV/2OSBN	2
PHBV/3OSBN	3
**SAINT GOBAIN (PHPP325B)**	
PHBV/0.5OSFBN	0.5
PHBV/1FBN	1
PHBV/1OSFBN	1
PHBV/2OSFBN	2
PHBV/3OSFBN	3

**Table 2:** (020)/(110) relative intensity ratios of nanocomposites.

(020)/(110)	
PHBV	1.42
PHBV/0.5OSBN	3.17
PHBV/1OSBN	3.40
PHBV/2OSBN	4.58
PHBV/3OSBN	2.56
PHBV/0.5OSFBN	4.98
PHBV/1OSFBN	6.72
PHBV/2OSFBN	4.70
PHBV/3OSFBN	3.76

**Table 3:** TG results of nanocomposites.

Sample	T_i_ (°C)	T_10_ (°C)	T_50_ (°C)	T_max_ (°C)	Char (%)
**PHBV**	234.45	243.50	256.04	275.08	1.81
**PHBV/0.5OSBN**	251.90	271.35	283.39	293.90	1.82
**PHBV/1BN**	250.30	271.97	282.97	292.30	2.03
**PHBV/1OSBN**	252.70	270.67	282.10	294.70	2.23
**PHBV/2OSBN**	253.50	271.53	282.73	295.50	2.88
**PHBV/3OSBN**	254.30	271.06	282.30	295.50	3.97
**PHBV/0.5OSFBN**	248.92	269.00	279.45	289.44	2.17
**PHBV/1FBN**	247.09	268.18	278.92	289.54	2.53
**PHBV/1OSFBN**	251.10	267.82	278.37	289.90	2.62
**PHBV/2OSFBN**	251.90	269.40	279.50	291.06	3.06
**PHBV/3OSFBN**	253.45	270.17	280.11	294.17	3.75

**Table 4:** DSC results of nanocomposites.

Sample	First Heating				Cooling		Second Heating		
	T_m1_ (°C)	T_m2_ (°C)	ΔH_m1_ (j/g)	X_c_ (%)	T_c1_ (°C)	ΔH_c_ (j/g)	T_m1_ (°C)	ΔH_m2_ (j/g)	X_c_ (%)
**PHBV**	170	173	88	60	122	86	171	100	68
**PHBV/0.5OSBN**	169	174	87	60	122	88	170	98	67
**PHBV/1BN**	171	-	81	57	124	84	172	95	65
**PHBV/1OSBN**	166	173	86	60	121	91	168	100	69
**PHBV/2OSBN**	171	-	86	60	122	87	170	100	70
**PHBV/3OSBN**	168	175	83	56	123	81	164	90	64
**PHBV/0.5OSFBN**	170	175	87	60	123	89	171	99	68
**PHBV/1FBN**	171	-	87	60	125	89	171	100	69
**PHBV/1OSFBN**	169	173	89	61	122	90	169	102	71
**PHBV/2OSFBN**	170	-	85	59	124	84	172	95	67
**PHBV/3OSFBN**	171	-	82	58	124	81	172	91	65

**Table 5:** Mechanical models [64,65].

Model	Array Type	Formula
Halpin–Tsai Model	Random array	$\begin{array}{c} \frac{E_{c}}{E_{m}}=\frac{3}{8}\left(\frac{1+\xi \eta_{L}\phi_{f}}{1-\eta_{L}\phi_{f}}\right)+\frac{5}{8}\left(\frac{1+2\eta_{T}\phi_{f}}{1-\eta_{T}\phi_{f}}\right)\\ \eta_{L}=\frac{\left(\frac{E_{f}}{E_{m}}\right)-1}{\left(\frac{E_{f}}{E_{m}}\right)+\xi }\\ \eta_{T}=\frac{\left(\frac{E_{f}}{E_{m}}\right)-1}{\left(\frac{E_{f}}{E_{m}}\right)+2} \end{array}$
Hui–Shia Model	Regular array	$\begin{array}{c} \frac{E_{c}}{E_{m}}=\frac{1}{1-\frac{\phi_{f}}{4}\left(\frac{1}{\xi }+\frac{3}{\xi +\Lambda }\right)}\\ \xi =\phi_{f}+\frac{E_{m}}{E_{f}-E_{m}}+3\left(-\phi_{f}\right)\left(\frac{\left(1-g\right)\alpha^{2}-\frac{g}{2}}{\alpha^{2}-1}\right)\\ g=\frac{\pi }{2}\alpha \\ \Lambda =\left(1-\phi_{f}\right)\left(\frac{3\left(\alpha^{2}+0,25\right)g-2\alpha^{2}}{\alpha^{2}-1}\right) \end{array}$

**Table 6:** Deviation (%) in E_c_/E_m_ of nanocomposites for mechanical models.

Sample	Halpin–Tsai Deviation (%)	Hui–Shia Deviation (%)
PHBV/0.5OSBN	2.480	1.743
PHBV/1OSBN	3.447	4.829
PHBV/2OSBN	3.575	6.306
PHBV/3OSBN	9.353	4.757
PHBV/0.5OSFBN	5.578	6.730
PHBV/1OSFBN	13.473	15.560
PHBV/2OSFBN	6.133	1.123
PHBV/3OSFBN	7.460	0.011
